# Prioritization and comprehensive analysis of genes related to major depressive disorder

**DOI:** 10.1002/mgg3.659

**Published:** 2019-04-09

**Authors:** Yi Liu, Pengfei Fan, Shiyuan Zhang, Yidan Wang, Dan Liu

**Affiliations:** ^1^ ICU First Teaching Hospital of Tianjin University of Traditional Chinese Medicine Tianjin P.R. China; ^2^ Organ Transplant Center Tianjin First Central Hospital Tianjin P.R. China; ^3^ Clinical Practice Teaching Center Tianjin University of Traditional Chinese Medicine Tianjin P.R. China; ^4^ Acupuncture Department First Teaching Hospital of Tianjin University of Traditional Chinese Medicine Tianjin P.R. China

**Keywords:** gene ontology, KEGG pathway, major depressive disorder, multi‐data‐source based prioritization

## Abstract

**Background:**

Major depressive disorder (MDD) is a serious mental health problem in modern society, which is difficult to identify and diagnose in the early stages. Despite strong evidence supporting the heritability of MDD, progresses in large‐scale and individual genetic studies remain preliminary.

**Methods:**

In this study, a multi‐data source‐based prioritization (MDSP) method was proposed, and an appropriate threshold was determined for the optimization of depression‐related genes (DEPgenes). Analyses on Gene Ontology biological processes, KEGG pathway and the specific pathway crosstalk network were further proposed.

**Results:**

A total of 143 DEPgenes were identified and the MDD‐specific network was constructed for the pathogenesis investigation and therapeutic methods development of MDD. Comparing with existing research strategies, the genetic optimization and analysis results were confirmed to be reliable. Finally, the pathway enrichment and crosstalk analyses revealed two unique pathway interaction modules that were significantly enriched with MDD genes. The related core pathways of neuroactive ligand‐receptor interaction and dopaminergic synapse supported the neuropathology hypothesis of MDD. And the pathways of serotonergic synapse and morphine addiction indicated the mechanism of drug addiction caused by serotonin used in the treatment.

**Conclusions:**

This work provided a reference for the study of MDD, although future validation by extensive experimentation is still required.

## 1 INTRODUCTION

Major depressive disorder (MDD) is a severe psychiatric disease with high morbidity and mortality worldwide (Culpepper, Lam, & McIntyre, 2017). This growing recognition of the public health burden has led to the development of depression detection and treatment. However, novel interventions of depression are still hindered by a limited understanding of the neurobiological mechanisms (Bayes & Parker, 2018). The efforts to clarify this biology through common or rare variant association studies seemed to be unsuccessful with the lack of distinct understanding of heterogeneity and absence of a biological gold‐standard diagnosis (Krystal & State, 2014). Nowadays, strong shreds of heritability evidence of mental diseases have been revealed (Alnaes et al., 2018; Pain et al., 2018), which attracted the studies on the generation of numerous genetic and genomic datasets in MDD studies.

During the past decade, rapid advances in high throughput technologies have helped investigators, aiming to uncover disease causal genes and their actions in complex diseases. Specifically, in psychiatric genetics, there have been numerous datasets from different platforms or sources such as association studies, including genome‐wide association studies, genome‐wide linkage scans, microarray gene expression, and copy number variation (Michaelson, 2017). Large‐scale and individual genetic studies revealed various polymorphisms and overexpression of certain genes in patients presenting with depressive symptoms (Lacerda‐Pinheiro et al., 2014; Milanesi et al., 2015). Zhang's group has found that, increased 5‐HT1A expression inversely correlated with 5‐HT activity via a negative feedback mechanism (Zhang et al., 2014). Moreover, HPA axis hyperactivity was reported as a trigger of MDD due to findings of GR and mineralocorticoid receptor dysfunction in depressed patients (Pariante & Lightman, 2008). However, a pervasive limitation in the existing research is the inherent heterogeneity in MDD studies, which impacts the validity of biomarker data (Young et al., 2016). Thus it is still necessary to simplify these depression‐related candidate genes to an optimal set for the subsequent biological experiments. Moreover, the incompletion of information resources used in existing calculation and the fixed screening threshold of corresponding online tools also result in arbitrarily preferred results and lower reliability.

In this study, gene information from multiple sources (including OMIM, Phenolyzer, GeneCards and GLAD4U) were integrated and analyzed for MDD. A multi‐data‐source based prioritization (MDSP) was proposed and an appropriate threshold was determined for the optimization of depression‐related genes (DEPgenes). Finally, the acquired genes which were significantly related to depression (DEPgenes) were verified by the receiver operating characteristic (ROC) curve and functional and pathway enrichment analysis. Our work demonstrated a practical framework for complex disease candidate gene analysis, which is of great significance for the comprehensive functional assessment of optimized pathogenic genes.

## 2 MATERIALS AND METHODS

### 2.1 MDD candidate genes and optimizing process

OMIM (www.omim.org), which provides vast repositories of rich clinical and genetic knowledge, was considered as a core gene database in this study. For association studies, the susceptibility genes were retrieved by searching all human genetic association studies deposited in Phenolyzer (phenolyzer.usc.edu), GeneCards (www.genecards.org) and GLAD4U (bioinfo.vanderbilt.edu/glad4u), which used as training gene categories. However, the background information of the dataset‐related patients is not provided in the database. For all the genes collected, genes presented in a certain training category were assigned a score of 1 point; otherwise, 0 was assigned. Thus, a gene could be represented by a vector of three elements, with each element being 1 or 0. When a gene showed up in all the training categories, all the elements in the vector would be 1's; on the other hand, a gene had at least one element being 1. For each training category, a weight was assigned to measure the category's reliability. A combined score derived from the category‐specific weight and gene score in the corresponding category was adopted to measure the correlation between a gene and the phenotype. All the candidate genes were ranked by their combined scores computed from their scores corresponding to the categories and the optimal weights. The combined scores were calculated by equation 1:

(1) $$S_{\text{Combined}}=\sum_{i=1}^{N}w_{i}\times {\text{Score}}_{i}$$

where *i* was the training category index, *N* = 3, *W_i_* was the corresponding weight of category*_i_*, and Score*_i_* and was equal to 1 when a gene showed up in category*_i_*; otherwise, Score*_i_*=0.

The combined score of a gene depends on its score from each training category and the corresponding weight value. In order to prioritize the genes collected so that the genes more likely correlated with MDD can be ranked higher in the list, a suitable weight for each training category needs to be determined. In this study, the following procedure was adopted:

1. Randomly selecting weight value between 0 and 1.0 for each training category and normalizing the weight matrix (consisted of the three weights) to have a sum of 1;
2. Calculating the combined score S for all genes by equation 1 and ranking all genes according to their combined scores;
3. Calculating ratio *R*: calculating the proportion *k* of core genes known to be related to MDD selected from OMIM in the top 3% of all candidate genes and *R* = *k*/23;
4. Reallocate values into the weight matrix and keeping the weight matrix to have a sum of 1.
5. Calculating ratio *R* after obtaining the new score *S* and ranking of all candidate genes;
6. Repeating steps 2–5 until no larger *R* can be found, and then the weight matrix obtained is the optimal weight matrix.

### 2.2 Evaluation of genetic optimizing results

The ROC curve was employed to assess the discrimination capability of the classifiers proposed in this study. ROC curves represent the performance of a classifier without taking into consideration class distribution or error overheads. And the classification success is then calculated by area under ROC curve (AUC) (Wray, Yang, Goddard, & Visscher, 2010). When the ROC curve deviated from the diagonal, i.e. the AUC value was close to 1, the verified method was evaluated as better reliability.

### 2.3 Functional and pathway enrichment tests

The relation of the prioritized genes with MDD was evaluated by analyzing the Gene Ontology (GO) biological processes or biochemical pathways enriched in these genes. The Database for Annotation, Visualization, Integration and Discovery (DAVID, david-d.ncifcrf.gov) was used for GO term enrichment analysis, followed by the correction of multiple testing using the Benjamini & Hochberg (BH) method. And the biological processes (BP) term was considered as significantly enriched with a cutoff of PBH < 0.01. In addition, KEGG pathway analysis was performed by WebGestalt online tool (www.webgestalt.org) (Wang, Vasaikar, Shi, Greer, & Zhang, 2017) and PBH < 0.05 was set as the cutoff criterion.

### 2.4 Pathway crosstalk

The pathway crosstalk analysis was performed to further investigate the interactions of significantly enriched pathways of optimized MDD‐related genes. Two pathways are considered to crosstalk if they share a proportion of DEPgenes. Two measurements were introduced to computationally indicate the overlap of a pair of pathways: Overlap coefficient (OC) = $\frac{\left|A⋂B\right|}{\min\left(\left|A\right|,\left|B\right|\right)}$ and Jaccard coefficient (JC) = $\frac{\left|A⋂B\right|}{\left|A⋃B\right|}$, where *A* and *B* denote the number of DEPgenes in the two pathways, respectively. The averages of OC and JC were calculated to reflect the overlap degree between pairs of pathways. And the crosstalk results were visualized by Cytoscape (Uzoma et al., 2018).

### 2.5 Depression‐specific network and cluster analysis by Cytoscape

To construct a depression‐specific network, the DEPgenes were imported into the STRING (string-db.org). The information on gene interaction was extracted and used to form a specific network. Module cluster analysis of the depression‐specific network was performed using the MCODE plug‐in in Cytoscape. Besides, to verify the nonrandomness of the obtained depression‐specific network, the following verification steps were performed:

1. Random network generation: generating 1,000 random networks which had the same node and interaction numbers as the depression‐specific network using Erdos‐Renyi model in an igraph package of R software;
2. Calculating the average shortest path distance (SPD) and average clustering coefficient (CC) of all the random networks, respectively.
3. Statistics: Calculating the number of the random networks that have shorter SPD than MDD‐specific network and the number of random network that have higher CC than MDD‐specific network, which denoted as ND and NC, respectively.
4. Calculating the experience *p*‐value: PD = ND/1,000 and PC = NC/1,000, which should reflect the significance of nonrandomness of MDD‐specific network.

## 3 RESULTS

### 3.1 Collection of MDD candidate and core genes

**Figure 1:**
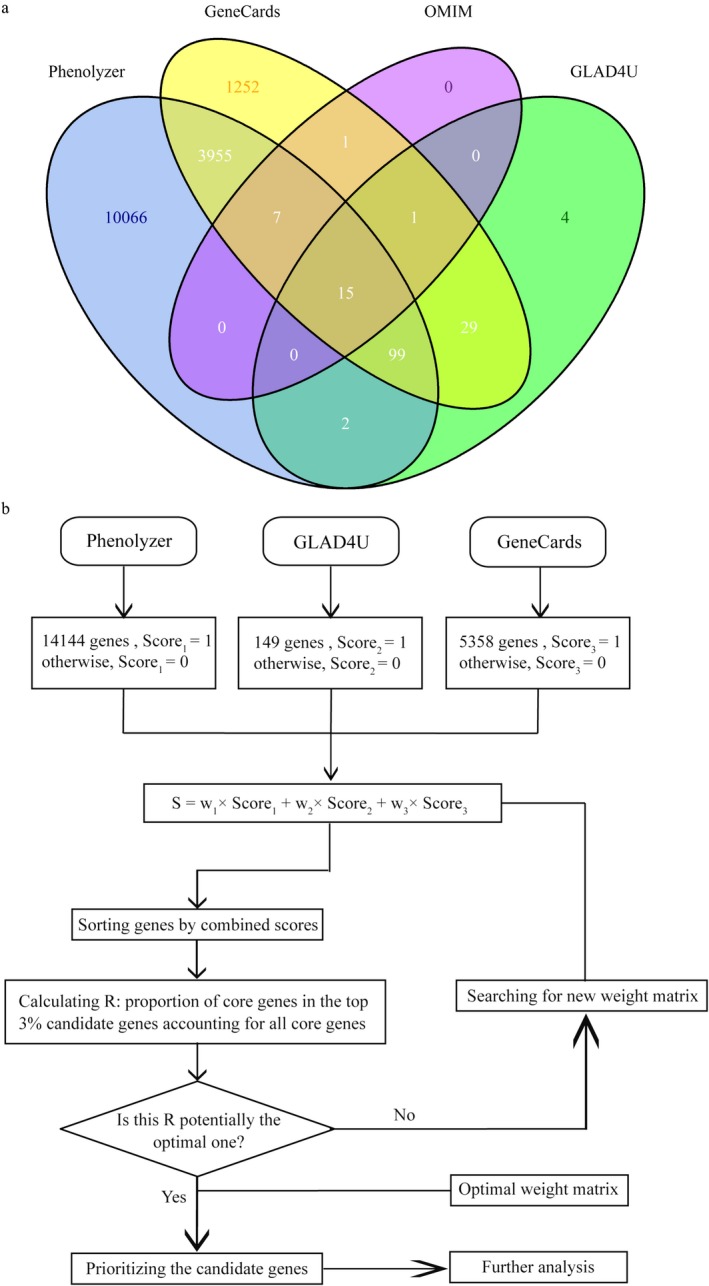
Overview of gene prioritization method. (a) Venn diagram of major depressive disorder (MDD)‐related candidate genes collected from different sources; (b) The flow chart for MDD‐related genes prioritization

A total of 23 genes were collected from OMIM (Table 1), which were regarded as core genes. Besides, 14,144 genes from Phenolyzer, 5,358 genes from GeneCards and 149 genes from GLAD4U were collected regarded as MDD candidate genes. These genes were collected from multi‐source, and each gene is showed up in a certain source in Figure 1a. MDSP was proposed and an appropriate threshold was determined for the optimization of MDD candidate genes. As the optimization algorithm flow chart of MDD candidate genes shown in Figure 1b, when a gene shows up in a certain training category, a score of 1 point is assigned; otherwise, 0 is assigned. Each of the four categories has a weight value, which is determined by the optimization algorithm as described in the "Material and Methods" section. The genes are ranked by their combined scores computed from scores of three training categories and their weights. Genes are ranked and prioritized by their combined scores, and further analysis is performed for the selected genes.

**Table 1:** Major depressive disorder core genes collected from OMIM

Gene symbol	MIM ID	Gene symbol	MIM ID
*MDD1*	608516	DRD4	608516
*MDD2*	608516	TPH1	608516
FKBP5	608516	HTR2C	608516
TPH2	608516	HTR1D	608516
HTR2A	608516	HTR1B	608516
CALCA	608516	MAOB	608516
DUSP1	608516	SLC6A4	608516
MTHFR	608516	BCR	608516
*CREB1*	608516	PER3	608516
HSP90AA1	608516	APAF1	608520
CHRM2	608516	SLC6A15	608520
TOR1A	608516		

### 3.2 Optimization and evaluation of MDD candidate genes

**Figure 2:**
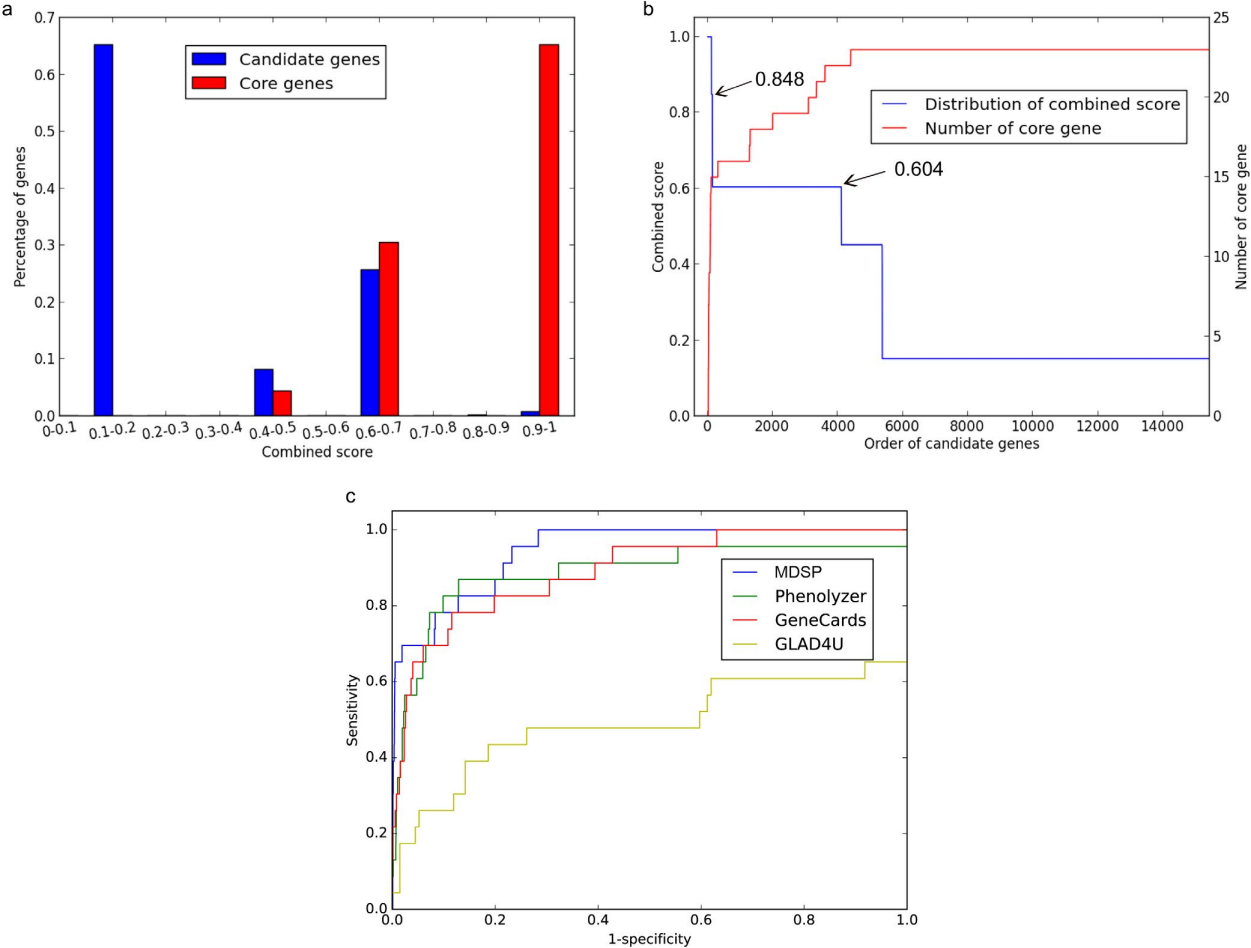
Optimization and evaluation of MDD candidate genes. (a) Distribution of the combined scores of all candidate genes and the core genes. The percentage of each histogram bin is measured by the genes with scores falling in the bin divided by the total number of candidate genes or the number of the core genes; (b) The distribution of the combined scores of the candidate genes. The genes are ranked by their combined scores. The *x*‐axis is the order of the candidate genes. The *y*‐axis on the left side is the combined score of the candidate genes, and the *y*‐axis on the right side is the number of core genes with higher combined score. (c) ROC curve of different prioritization tools. MDD: major depressive disorder; ROC: receiver operating characteristic

The combined scores of all candidate genes were calculated based on the optimal weight matrix and the candidate gene score in each source. The MDD candidate genes were ranked according to the combined scores. The gene list and the combined scores distribution of core genes and all candidate genes optimized by our process are shown in Figure 2a. Most of the core genes with higher combined scores appeared in front of the sorted list, and only several appeared in the posterior position, indicating that the distribution of the candidate genes' combined scores was in line with our expectations.

From Figure 2b, it was inferred that, the score drops quickly from 1.0 to about 0.848 and then drops to about 0.604; after that, the combined scores decrease slowly. Such a distribution indicated that a relatively small number of genes have higher combined scores, while the majority of genes has moderate or small scores. With a threshold of 0.848, 65.2% of the core genes (15/23) were contained. Although with a threshold of 0.604, 95.7% of the core genes (22/23) could be contained, the number of selected candidate genes would also dramatically increase to 4,105. As the smaller the comprehensive score was, the higher the false positive rate of the prioritized gene was, 143 DEPgenes were identified with a threshold of 0.848 (Table S1).

Finally, the reliability of our method for prioritizing MDD candidate genes was compared with Phenolyzer, GeneCards and GLAD4U through ROC curve. As a result, AUC of MDSP (0.944) is the largest followed by GeneCards (0.893) and Phenolyzer (0.888), and GLAD4U had the smallest AUC value (0.490), which indicated that the results of the MDSP optimization were the best.

### 3.3 GO enrichment analysis

To explore specific functional features of the 143 DEPgenes, GO enrichment analysis was performed using DAVID. Seventy‐two biological processes (BP terms) which related to synaptic transmission, neurodevelopment and drug reaction were significantly enriched in DEPgenes (Table 2). The GO terms related to synaptic transmission included synaptic transmission, regulation of synaptic transmission, positive regulation of synaptic transmission and negative regulation of synaptic transmission. The GO terms related to nerve signal transduction included second‐messenger‐mediated signaling, regulation of transmission of nerve impulse, cell surface receptor linked signal transduction, G‐protein coupled receptor protein signaling pathway and glutamate signaling pathway. The GO terms related to neurotransmitter, such as regulation of neurotransmitter levels, regulation of neurotransmitter transport, regulation of neurotransmitter uptake, regulation of catecholamine secretion, regulation of dopamine secretion and regulation of glutamate secretion, while that related to drug reaction (response to tropane, response to cocaine, response to amphetamine and response to histamine) and learning or memory were also significantly enriched.

**Table 2:** Significantly enriched BP terms of the 143 DEPgenes

GO terms	Biological process	No. of genes	*p*‐value	PBH
GO:0007268	Synaptic transmission	36	1.24E‐32	1.02E‐29
GO:0019932	Second‐messenger‐mediated signaling	22	5.46E‐17	2.25E‐14
GO:0030808	Regulation of nucleotide biosynthetic process	16	4.37E‐15	1.19E‐12
GO:0050804	Regulation of synaptic transmission	17	5.37E‐15	1.10E‐12
GO:0006140	Regulation of nucleotide metabolic process	16	9.79E‐15	1.61E‐12
GO:0051969	Regulation of transmission of nerve impulse	17	1.89E‐14	2.60E‐12
GO:0031644	Regulation of neurological system process	17	3.56E‐14	4.21E‐12
GO:0007166	Cell surface receptor linked signal transduction	46	8.23E‐14	8.49E‐12
GO:0045761	Regulation of adenylate cyclase activity	14	3.60E‐13	3.30E‐11
GO:0007186	G‐protein coupled receptor protein signaling pathway	33	2.50E‐11	2.06E‐09
GO:0051046	Regulation of secretion	16	3.56E‐11	2.67E‐09
GO:0001505	Regulation of neurotransmitter levels	11	8.09E‐11	5.57E‐09
GO:0051952	Regulation of amine transport	9	1.12E‐10	7.10E‐09
GO:0031280	Negative regulation of cyclase activity	10	3.17E‐10	1.87E‐08
GO:0051350	Negative regulation of lyase activity	10	3.17E‐10	1.87E‐08
GO:0007611	Learning or memory	12	8.15E‐10	4.49E‐08
GO:0051050	Positive regulation of transport	15	1.55E‐09	8.01E‐08
GO:0014073	Response to tropane	7	4.54E‐09	2.21E‐07
GO:0042220	Response to cocaine	7	4.54E‐09	2.21E‐07
GO:0051940	Regulation of catecholamine uptake during transmission of nerve impulse	5	1.66E‐08	7.64E‐07
GO:0051588	Regulation of neurotransmitter transport	7	3.69E‐08	1.61E‐06
GO:0051580	Regulation of neurotransmitter uptake	5	4.96E‐08	2.05E‐06
GO:0007242	Intracellular signaling cascade	29	1.45E‐07	5.70E‐06
GO:0009712	Catechol metabolic process	7	2.05E‐07	7.70E‐06
GO:0006584	Catecholamine metabolic process	7	2.05E‐07	7.70E‐06
GO:0006576	Biogenic amine metabolic process	9	7.77E‐07	2.79E‐05
GO:0014059	Regulation of dopamine secretion	5	1.06E‐06	3.65E‐05
GO:0051047	Positive regulation of secretion	9	1.89E‐06	6.25E‐05
GO:0051954	Positive regulation of amine transport	5	3.16E‐06	1.00E‐04
GO:0030003	Cellular cation homeostasis	12	3.99E‐06	1.22E‐04
GO:0001662	Behavioral fear response	5	4.28E‐06	1.26E‐04
GO:0031281	Positive regulation of cyclase activity	7	4.80E‐06	1.37E‐04
GO:0006939	Smooth muscle contraction	6	4.96E‐06	1.37E‐04
GO:0001964	Startle response	5	5.68E‐06	1.51E‐04
GO:0050806	Positive regulation of synaptic transmission	6	5.78E‐06	1.49E‐04
GO:0051349	Positive regulation of lyase activity	7	5.89E‐06	1.47E‐04
GO:0008306	Associative learning	5	7.38E‐06	1.79E‐04
GO:0015844	Monoamine transport	5	7.38E‐06	1.79E‐04
GO:0051971	Positive regulation of transmission of nerve impulse	6	8.89E‐06	2.10E‐04
GO:0043269	Regulation of ion transport	8	1.10E‐05	2.52E‐04
GO:0014075	Response to amine stimulus	6	1.16E‐05	2.59E‐04
GO:0031646	Positive regulation of neurological system process	6	1.16E‐05	2.59E‐04
GO:0008217	Regulation of blood pressure	8	1.17E‐05	2.55E‐04
GO:0050433	Regulation of catecholamine secretion	5	1.19E‐05	2.51E‐04
GO:0001975	Response to amphetamine	5	1.19E‐05	2.51E‐04
GO:0050805	Negative regulation of synaptic transmission	5	1.81E‐05	3.74E‐04
GO:0044106	Cellular amine metabolic process	12	2.24E‐05	4.52E‐04
GO:0042053	Regulation of dopamine metabolic process	4	2.44E‐05	4.79E‐04
GO:0055082	Cellular chemical homeostasis	13	3.46E‐05	6.65E‐04
GO:0042069	Regulation of catecholamine metabolic process	4	3.63E‐05	6.82E‐04
GO:0010959	Regulation of metal ion transport	7	3.70E‐05	6.79E‐04
GO:0007215	Glutamate signaling pathway	5	3.74E‐05	6.71E‐04
GO:0051970	Negative regulation of transmission of nerve impulse	5	3.74E‐05	6.71E‐04
GO:0060134	Prepulse inhibition	4	5.16E‐05	9.06E‐04
GO:0060191	Regulation of lipase activity	7	5.55E‐05	9.54E‐04
GO:0031645	Negative regulation of neurological system process	5	5.95E‐05	1.00E‐03
GO:0050801	Ion homeostasis	13	7.05E‐05	1.16E‐03
GO:0032309	Icosanoid secretion	4	7.06E‐05	1.14E‐03
GO:0050482	Arachidonic acid secretion	4	7.06E‐05	1.14E‐03
GO:0007632	Visual behavior	5	8.98E‐05	1.43E‐03
GO:0014048	Regulation of glutamate secretion	4	1.21E‐04	1.88E‐03
GO:0033238	Regulation of cellular amine metabolic process	4	1.21E‐04	1.88E‐03
GO:0034776	Response to histamine	3	1.76E‐04	2.70E‐03
GO:0046717	Acid secretion	4	3.35E‐04	5.03E‐03
GO:0048699	Generation of neurons	14	3.51E‐04	5.17E‐03
GO:0015909	Long‐chain fatty acid transport	4	5.38E‐04	7.76E‐03
GO:0019614	Catechol catabolic process	3	5.82E‐04	8.26E‐03
GO:0015718	Monocarboxylic acid transport	5	5.87E‐04	8.19E‐03
GO:0032102	Negative regulation of response to external stimulus	5	5.87E‐04	8.19E‐03
GO:0010648	Negative regulation of cell communication	9	6.57E‐04	9.01E‐03
GO:0022008	Neurogenesis	14	6.97E‐04	9.39E‐03
GO:0043271	Negative regulation of ion transport	4	7.08E‐04	9.39E‐03

DEPgenes: depression‐related genes; GO: gene ontology.

### 3.4 Crosstalk among significantly enriched pathways

**Figure 3:**
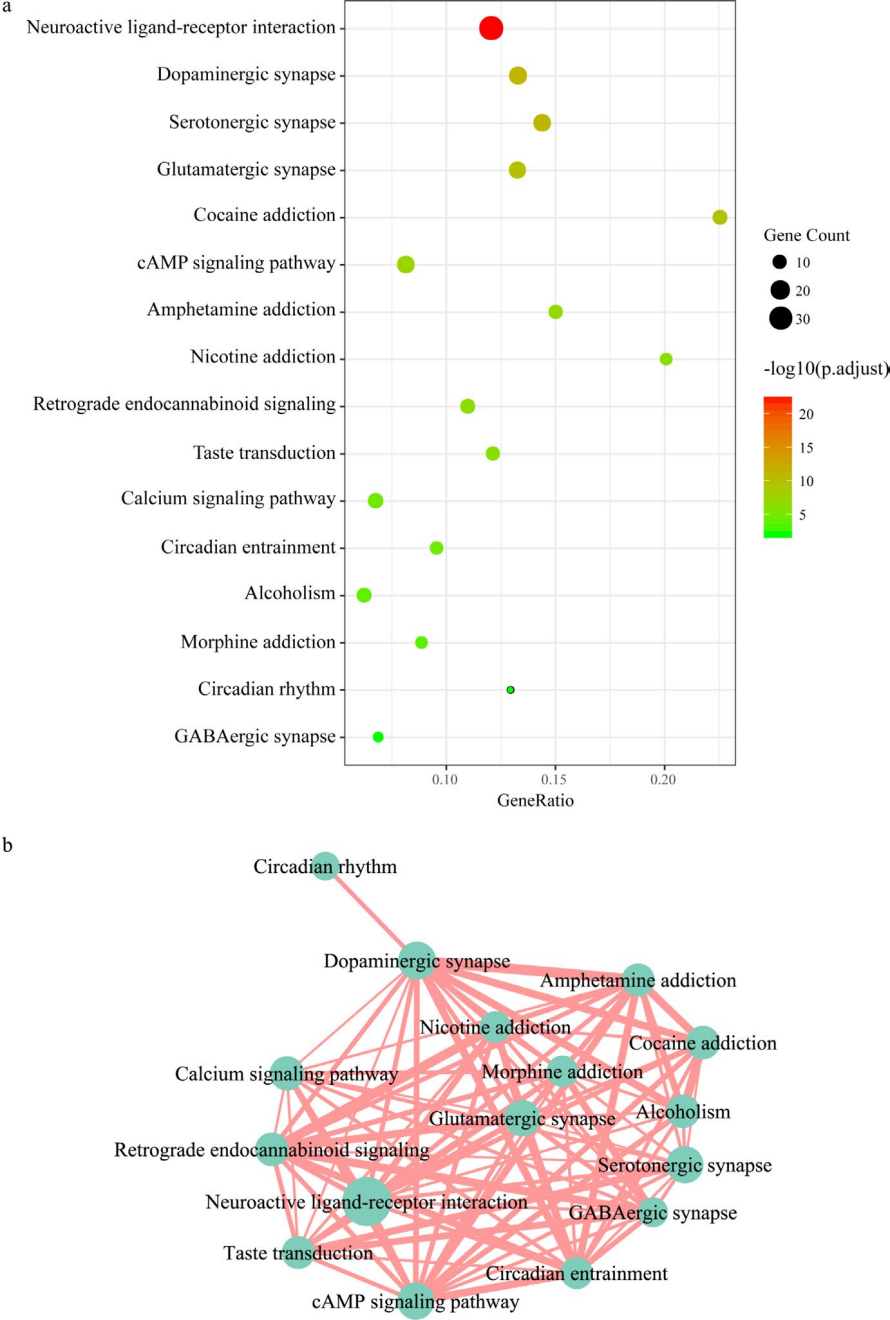
KEGG pathway enrichment analysis of DEPgenes. (a) Significantly enriched KEGG pathways of DEPgenes. The abscissa GeneRatio was the ratio of DEPgenes mapped to a KEGG pathway to the total number of genes in the pathway; (b) Visual crosstalk of KEGG pathways. The nodes size represented the number of DEPgenes contained in the pathway. The larger the node was, the more DEPgenes were included. The width of the edge indicated the overlapping degree of genes contained in two pathways. DEPgenes: depression‐related genes

Since abundant genes and pathways seemed to be involved in MDD, a pathway crosstalk analysis was performed to deeply investigate the relationship between the pathways. As shown in Figure 3a, 16 significantly enriched pathways were identified, including nervous system pathways, such as Dopaminergic synapse, serotonergic synapse, glutamatergic synapse, retrograde endocannabinoid signaling and GABAergic synapse. Besides, the pathways related to drug addiction (cocaine addiction, amphetamine addiction, nicotine addiction, alcoholism and morphine addiction), signal transduction (cAMP signaling pathway, taste transduction and calcium signaling pathway) were enriched. Interestingly, the environmental adaptation processes (circadian entrainment and circadian rhythm) were also involved in the DEPgenes' pathways. In Figure 3b, it was clear that the significantly enriched pathways were clustered into a module which was relevant to the pathogenesis of neurological diseases.

### 3.5 MDD‐specific networks

**Figure 4:**
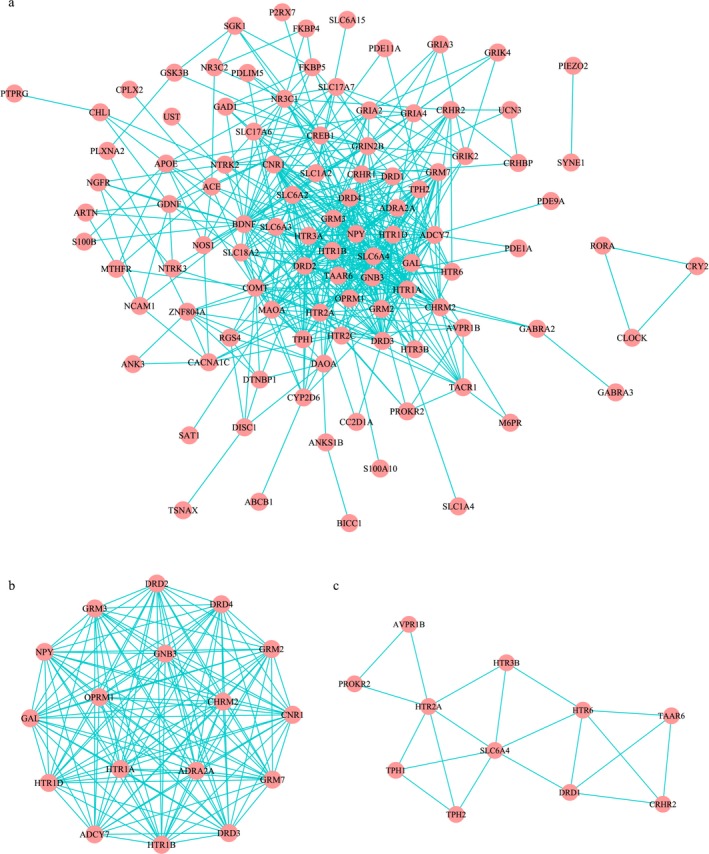
MDD‐specific network analysis. (a) The specific network of MDD; (b and c) Module Cluster analyses by MCODE. MDD: major depressive disorder

The information on gene interaction was extracted from the STRING database and used to form a specific network (Figure 4a). To test nonrandomness of the MDD‐specific network, we generated 1,000 random networks with same node and edge number with MDD‐specific network and compared their SPD and CC. As a result, the average SPD of these random networks was 3.4, which was significantly larger than that of the MDD‐specific network with an SPD of 2.5, PD < 0.001. Meanwhile, the CC of random networks was 0.1, which was significantly smaller than that of the MDD‐specific networks with a CC of 0.5 (PC < 0.001). So, the nonrandomness of the MDD‐specific network could be inferred. Furthermore, two modules were identified by the modular cluster analysis of MDD‐specific networks (Figure 4b,c). KEGG pathway analysis of genes contained in Figure 4b indicated significantly enriched pathways of neuroactive ligand‐receptor interaction, dopaminergic synapse and morphine addiction. For genes contained in Figure 4c, the serotonergic synapse was the most significantly enriched pathway.

## 4 DISCUSSION

Drug therapy is still the preferred current clinical treatment for MDD. The most widely used antidepressant drugs are selective serotonin reuptake inhibitors (SSRIs), including fluoxetine, citalopram, and sertraline, which can significantly improve cognitive function of MDD patients (Jakubovski, Varigonda, Freemantle, Taylor, & Bloch, 2016). However, current antidepressant drugs used clinically bring lots of adverse reactions, such as xerostomia, constipation, drowsiness, obesity, cardiotoxicity, and drug withdrawal (Fava, Gatti, Belaise, Guidi, & Offidani, 2015; Hieronymus, Emilsson, Nilsson, & Eriksson, 2016). The lack of approaches on early identification and intervention of MDD patients limits the establishment of safe and effective individualized treatment (Duman, Aghajanian, Sanacora, & Krystal, 2016). Although numerous reports of susceptibility genes or loci to MDD have been reported previously, no disease causal genes and therapeutic target genes were confirmed (Rao et al., 2016). Thus, it is important to reduce the data noise and prioritize candidate genes from multiple datasets and then explore their functional relationships for further validation (Jia, Kao, Kuo, & Zhao, 2011).

In this study, we presented a complete process to collect large‐scale genotypic data on MDD from different sources, and provided optimization and comprehensive analyses for the exploration of the pathogenesis and treatment of depression. Twenty‐three DEPgenes from OMIM, 14,144 DEPgenes from Phenolyzer, 5,358 DEPgenes from GeneCards and 149 DEPgenes from GLAD4U were collected and optimized for further analyzation. MDSP was proposed and an appropriate threshold was determined for the optimization of MDD‐related genes. One hundred and forty‐three DEPgenes were identified and used for additional functional and pathway enrichment analyses. Most of these genes, such as *PCDH9*, *MDD1*, *MDD2*, *CREB1* and *DISC1*, have been identified to be associated with MDD (Cacabelos, Torrellas, & Fernandez‐Novoa, 2016; Xiao et al., 2018), and some of them (e.g. TPH1, GRIN2B and MAOA) were also related to other mental disorders (van Donkelaar et al., 2017; Perlis, 2016; Tovilla‐Zarate et al., 2014). This indicated that our preferred solution designed was able to be utilized to get the expected data.

So far, the study of the pathogenesis of depression mainly focuses on the biological mechanisms, such as autophagy and apoptosis of nerve cells, neurotransmitter secretion disorders, immune inflammatory reactions, dysfunction of hypothalamus pituitary adrenal axis, and other biological mechanisms (Cattaneo et al., 2015; Menard, Hodes, & Russo, 2016; Smith, 2015). With functional enrichment analysis, a more specific functional pattern implicated in these DEPgenes was revealed. In this study, 72 GO BP terms and 16 KEGG pathways were identified to be significantly enriched. The terms related to synaptic transmission, nerve signal transduction, neurotransmitter and learning or memory reflected the pathogenesis of MDD, which was consistent with the literature reports. Interestingly, the BP term of drug reaction and the KEGG pathway of drug addiction were both enriched, indicating that the key requirement of avoiding drug dependence in MDD drug development and clinical treatment.

The occurrence and development of MDD involve complex biological processes, which is the result of a combination of multiple genes and environmental factors. Therefore, the study of the interactions between DEPgenes from the perspective of networks can provide insights into the pathogenesis of depression and contribute to the discovery of new drug targets. Thus, the network information on MDD was mined from the STRING database which contains experimental data, the PubMed abstract text database and results predicted by bioinformatics methods for specific analysis. Besides, applied bioinformatics methods in this process included gene adjacency, gene fusion, phylogenetic profiles, and gene co‐expression based on chip data. A comprehensive score was calculated with the weight matrix of these different methods determined by a scoring mechanism demonstrated above. Finally, the core pathways involved in MDD were shown in the module. The pathways of neuroactive ligand‐receptor interaction, dopaminergic synapse and morphine addiction are presented in Figure 4b. And as shown in Figure 4c, the serotonergic synapse seemed to be higher specificity than other pathways. From these results, we inferred that the drug addiction caused by serotonin used in the treatment of MDD might relate to the mechanism of morphine addiction.

The main problems that limit the development of a reliably viable MDD biomarker are the heterogeneity of depressive disorder pathophysiology, etiology, and study designs, which may bring in conflicting data. In this study, a systems biology framework for the genetic information collection, advanced function and pathway analyses for MDD was developed. A total of 143 DEPgenes were identified and the MDD‐specific network was constructed for the pathogenesis investigation and therapeutic methods development of MDD. Comparing with existing research strategies, the genetic optimization and analysis results were confirmed to be reliable. As most studies collected data from small samples sizes often consisting of fewer than 100 subjects, this study would contribute to improving the precision and generalizability of MDD‐related genes in these three databases. However, although this computational framework applied quantity of valuable information that required future validation by extensive experimental, it still provided a reference for the study of other complex disease.

## 5 ETHICS APPROVAL AND CONSENT TO PARTICIPATE

Not applicable.

## CONFLICT OF INTEREST

The authors declare no conflict of interest.

## AUTHORS' CONTRIBUTIONS

Yi Liu and Shiyuan Zhang conceived and designed the project, Pengfei Fan acquired the data, Yi Liu, Pengfei Fan and Yidan Wang analyzed and interpreted the data, Yidan Wang and Dan Liu wrote the paper. Shiyuan Zhang approved the final version.
