# Supplementary material for: Prioritization and comprehensive analysis of genes related to major depressive disorder
Source: Mol Genet Genomic Med. 2019 Apr 9;7(6):e659. doi: 10.1002/mgg3.659 (PMC6565567; doi:10.1002/mgg3.659)
Supplement: Supplementary file 1 [file MGG3-7-e659-s001.docx]

Table S1 The 143 DEPgenes identified with a threshold of 0.848.

| Gene Symbol | Combined Score | Gene Symbol | Combined Score | Gene Symbol | Combined Score |
| --- | --- | --- | --- | --- | --- |
| SP4 | 1.0 | WFS1 | 1.0 | S100B | 1.0 |
| ARTN | 1.0 | ZNF804A | 1.0 | ANK3 | 1.0 |
| PROKR2 | 1.0 | NCAM1 | 1.0 | MIR4495 | 1.0 |
| NTRK2 | 1.0 | TACR1 | 1.0 | SLC6A3 | 1.0 |
| NTRK3 | 1.0 | CRY2 | 1.0 | SLC6A2 | 1.0 |
| GRIN2B | 1.0 | CACNA1C | 1.0 | S100A10 | 1.0 |
| SLC18A2 | 1.0 | HTR6 | 1.0 | HTR1B | 1.0 |
| SLC17A6 | 1.0 | GSK3B | 1.0 | SYNE1 | 1.0 |
| SLC17A7 | 1.0 | RNF123 | 1.0 | NR3C1 | 1.0 |
| ADRA2A | 1.0 | P2RX7 | 1.0 | FKBP4 | 1.0 |
| PDE11A | 1.0 | GAD1 | 1.0 | CC2D1A | 1.0 |
| GPR50 | 1.0 | GRIK2 | 1.0 | OPRM1 | 1.0 |
| CNR1 | 1.0 | GRIK4 | 1.0 | AVPR1B | 1.0 |
| ANKS1B | 1.0 | COMT | 1.0 | PIEZO2 | 1.0 |
| APOE | 1.0 | M6PR | 1.0 | TSNAX | 1.0 |
| ADCY7 | 1.0 | BDNF | 1.0 | CRHBP | 1.0 |
| HTR2A | 1.0 | DRD1 | 1.0 | PLXNA2 | 1.0 |
| HTR2C | 1.0 | GNB3 | 1.0 | TUBAP | 1.0 |
| ACE | 1.0 | CRHR2 | 1.0 | MYT1L | 0.848 |
| MTHFR | 1.0 | DTNBP1 | 1.0 | LOC101928937 | 0.848 |
| PTPRG | 1.0 | DRD4 | 1.0 | LOC100130203 | 0.848 |
| CHL1 | 1.0 | DRD2 | 1.0 | LOC101928354 | 0.848 |
| NGFR | 1.0 | DRD3 | 1.0 | GLT8D1 | 0.848 |
| RORA | 1.0 | LIMD1-AS1 | 1.0 | DAOA | 0.848 |
| TPH1 | 1.0 | HTR3B | 1.0 | ISX-AS1 | 0.848 |
| PDE9A | 1.0 | HTR3A | 1.0 | FAM155A | 0.848 |
| MDD2 | 1.0 | CREB1 | 1.0 | LOC101927795 | 0.848 |
| MDD1 | 1.0 | CLOCK | 1.0 | HGC6.3 | 0.848 |
| MIR1202 | 1.0 | WDR26 | 1.0 | ID2B | 0.848 |
| FKBP5 | 1.0 | CRHR1 | 1.0 | EIF4A1P1 | 0.848 |
| CYP2D6 | 1.0 | GAL | 1.0 | LINC00276 | 0.848 |
| RGS4 | 1.0 | HTR1D | 1.0 | SEC14L5 | 0.848 |
| NR3C2 | 1.0 | HTR1A | 1.0 | LOC102723722 | 0.848 |
| SAT1 | 1.0 | EHD3 | 1.0 | LOC158435 | 0.848 |
| GDNF | 1.0 | GABRA2 | 1.0 | BICC1 | 0.848 |
| SGK1 | 1.0 | GRIA3 | 1.0 | LOC102723706 | 0.848 |
| PDE1A | 1.0 | SLC6A15 | 1.0 | PDLIM5 | 0.848 |
| CHRM2 | 1.0 | GRM7 | 1.0 | RPL21P24 | 0.848 |
| PCLO | 1.0 | GRM2 | 1.0 | LSAMP | 0.848 |
| GRIA2 | 1.0 | GRM3 | 1.0 | LOC101928516 | 0.848 |
| GRIA4 | 1.0 | UST | 1.0 | DCANP1 | 0.848 |
| SLC6A4 | 1.0 | TPH2 | 1.0 | HSPE1P1 | 0.848 |
| NOS1 | 1.0 | SLC1A4 | 1.0 | ITIH3 | 0.848 |
| MAOA | 1.0 | SLC1A2 | 1.0 | LOC101927056 | 0.848 |
| GABRA3 | 1.0 | CPLX2 | 1.0 | VENTXP1 | 0.848 |
| TAAR6 | 1.0 | GMIP | 1.0 | DISC1 | 0.848 |
| ABCB1 | 1.0 | MIR4789 | 1.0 | MTCL1P1 | 0.848 |
| NPY | 1.0 | UCN3 | 1.0 |  |  |
